# Assessment of Relative Accuracy of AHN-2 Laser Scanning Data Using Planar Features

**DOI:** 10.3390/s100908198

**Published:** 2010-09-01

**Authors:** Corné van der Sande, Sylvie Soudarissanane, Kourosh Khoshelham

**Affiliations:** Optical and Laser Remote Sensing, Department of Remote Sensing, Delft University of Technology, Kluyverweg 1, 2629HS Delft, The Netherlands; E-Mails: C.J.vanderSande@tudelft.nl (C.S.); S.S.Soudarissanane@tudelft.nl (S.S.)

**Keywords:** Airborne Laser Scanning, altimetry, accuracy assessment, strip adjustment, planar features, least-squares estimation

## Abstract

AHN-2 is the second part of the Actueel Hoogtebestand Nederland project, which concerns the acquisition of high-resolution altimetry data over the entire Netherlands using airborne laser scanning. The accuracy assessment of laser altimetry data usually relies on comparing corresponding tie elements, often points or lines, in the overlapping strips. This paper proposes a new approach to strip adjustment and accuracy assessment of AHN-2 data by using planar features. In the proposed approach a transformation is estimated between two overlapping strips by minimizing the distances between points in one strip and their corresponding planes in the other. The planes and the corresponding points are extracted in an automated segmentation process. The point-to-plane distances are used as observables in an estimation model, whereby the parameters of a transformation between the two strips and their associated quality measures are estimated. We demonstrate the performance of the method for the accuracy assessment of the AHN-2 dataset over Zeeland province of The Netherlands. The results show vertical offsets of up to 4 cm between the overlapping strips, and horizontal offsets ranging from 2 cm to 34 cm.

## 1. Introduction

Airborne Laser Scanning is an active optical range measurement technique that is used for acquiring accurate height data of the ground surface. The advantages of airborne laser scanning include high measurement accuracy, fast acquisition capability, and large spatial coverage. The Netherlands is the first country that was entirely covered by airborne laser altimetry measurements through the Actueel Hoogtebestand Nederland (AHN) project [1]. The second part of the national laser altimetry project AHN-2 is being carried out since 2007 by several companies, and will be completed in 2012. The new height model has an unprecedented spatial resolution: it has an average density of 10 points per square meter [2]. This unique high-resolution height model is used in a variety of applications, including water storage determination, subsidence studies, forest volume mapping, monitoring of coastal areas and 3D modeling of urban environments.

The airborne laser scanning measurements are acquired in multiple flight strips, and are independently georeferenced using data from on-board navigation sensors, *i.e.*, Global Navigation Satellite System (GNSS) and Inertial Navigation System (INS). The systematic and random errors introduced by the scanning mechanism as well as the navigation sensors accumulate in the final point cloud. This results in an offset and misalignment between data of overlapping flight strips. The quantification and elimination of the offset and misalignment between the overlapping strips is an important issue in airborne laser scanning, and is commonly referred to as strip adjustment. Moreover, the identification of the offsets and misalignments forms a major part of the accuracy assessment done by the end-user of the data. Therefore, fast and reliable methods for the adjustment of aerial laser strips are of particular interest for both the data providers and the end-users.

The error budget of airborne laser scanning has been a subject of extensive research [3–9]. In general, the errors originate from three main sources: (i) individual sensors (scanner, INS, GNSS) and their integration; (ii) properties of the target surface (geometry, reflectance); and (iii) post-processing (filtering, interpolation). Among these, the first source has a major impact on the accuracy of raw height data. The conventional approach to assessing the accuracy of laser strips is based on comparing the height on a number of tie points (corresponding points in two overlapping laser strips) or control points (ground truth measurements). Kilian *et al*. [6], Crombaghs *et al*. [10], and Hodgson and Bresnahan [7] describe the use of tie and control points for the accuracy assessment of airborne laser scanner data. The identification of point features in laser scanner data is however difficult, due to the discrete nature of the data and the limited spatial resolution. To allow the accurate measurement of control points, Csanyi and Toth [11] designed ground targets of a specific shape suitable for measurement in laser altimetry data. As another source of external measurements, Bretar *et al*. [12] used a photogrammetrically derived Digital Surface Model (DSM) to perform an adjustment of airborne laser strips.

An alternative approach to assessing the relative accuracy of laser altimetry data is the comparison of corresponding features in overlapping strips. Vosselman [13,14] estimated planimetric offsets between the strips using linear features such as ditches and roof ridge lines. The planimetric accuracy of laser altimetry data is generally lower than the altimetric accuracy [5], but is equally important, particularly in applications where mapping object boundaries is concerned. The use of corresponding linear features for the accuracy assessment has also been reported in a number of recent works [15–17]. Other previous works have demonstrated the use of corresponding surfaces for the evaluation of the errors in laser altimetry data. Ressl [18] presented a non-linear model for accuracy assessment of strip adjustment based on five parameters. Latypov [19] derived a generalized relative accuracy measure by comparing corresponding surfaces in the overlapping strips. Corresponding planar surfaces have been used for the estimation of vertical offsets between the strips [20], as well as the system calibration parameters [21,22]. Maas [23] estimated shift parameters between two overlapping or crossing strips by minimizing the distances between points in one strip and a triangulated mesh (TIN) of the other strip. Habib *et al*. [9,24] also minimized point-to-TIN distances iteratively, but estimated biases in the laser scanner system components. The drawback of the TIN-based methods is that the triangular patches do not necessarily always represent actual surfaces.

This paper proposes a new approach to strip adjustment and accuracy assessment of airborne laser scanner data by using planar features. The choice of planar features is particularly appropriate for the accuracy assessment of AHN-2 data over the Netherlands, because buildings with gable roof planes are present almost in all parts of the country, and also the high point density of the AHN-2 data is a determining factor in the increased reliability of the extracted planes. The basis of the proposed approach is that by evaluating the adjustment between the corresponding planes in two overlapping strips an estimation of the systematic and random errors in the data can be obtained. In the strip adjustment, a transformation is estimated between the strips by minimizing the distances between points in one strip and their corresponding planes in the other. The advantage of such an estimation model is twofold. First, incorporating a large number of reliable point-to-plane distance observations in a least-squares estimation model improves the precision of the estimated transformation parameters. Second, if two strips cannot be adjusted by only a 3D offset, an affine transformation can be estimated from which possible rotations between the strips can be derived. In addition, the estimation model is linear, which makes it independent of an initial approximation of the transformation parameters.

The paper proceeds with an overview of laser altimetry over the Netherlands and the status of the AHN-2 project in Section 2. Section 3 describes the proposed approach to strip adjustment, including the segmentation method for extracting planar segments, the robust plane fitting and the mathematical model for the estimation of strip adjustment parameters. The results of the accuracy assessment of the AHN-2 data over Zeeland province are presented in Section 4. The paper concludes with some remarks in Section 5.

## 2. Airborne Laser Altimetry over the Netherlands: The AHN-2 Project

**Figure 1.**
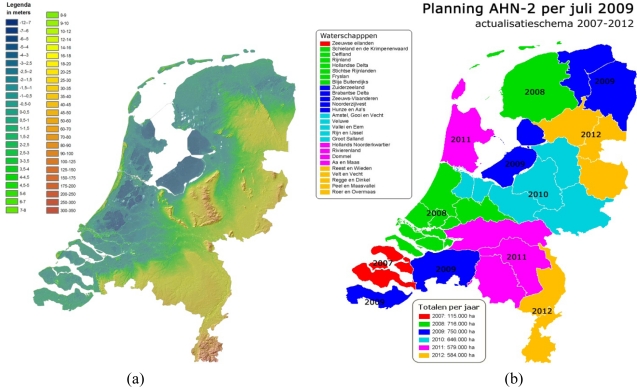
The AHN project. **(a)** the height model acquired during the first phase of the AHN project; **(b)** the acquisition plan of AHN-2.

The Actual Height model of the Netherlands (AHN) part 2 is a joint program between the ministry of water management (Rijkswaterstaat) and the 26 water boards conducted in the period from 2007 to 2012. Water boards are the oldest legislative bodies in the Netherlands responsible for monitoring dykes and managing water quantity and quality. The importance of a country-wide height model for these organisations is evident, as the Netherlands is a delta partly below the sea level. During the first phase of the AHN project between 1997 and 2003 the entire Netherlands was laser scanned to make a height model with an altimetric accuracy of 16 cm and a point density varying from 1 point per 16 m^2^ to 1 point per 32 m^2^. For the AHN-2 the requirements were tightened to achieve a higher accuracy and an increased point density. Figure 1 shows the first height model of the Netherlands, and the acquisition plan of the AHN-2.

While the AHN-2 is coordinated by the Waterschapshuis (the executive body of the Dutch water boards in the area of information and communication technology) the acquisition and quality control of the data are done by private companies. The first acquisition was done by Fugro over Zeeland province in the winter of 2007 using the FLI-MAP 400 laser scanner system mounted on a helicopter [25]. The FLI-MAP 400 system uses the Multiple Pulse in Air (MPiA) technique, which is the state of the art in airborne laser scanning technology. The MPiA technique allows the laser scanner to operate at a higher pulse frequency (375 KHz in the case of FLI-MAP 400) by emitting a second pulse before the reflection of the first one is received [26]. The result is a substantial increase in the maximum flying height and the coverage of each laser strip, but also an increased point density on the ground for a given flying height.

The planned specifications of the AHN-2 data include an average density of 10 points per m^2^ with 5 cm systematic error in height and 5 cm standard deviation. With such point density an object of 2 m by 2 m size can be correctly identified with a maximum planimetric error of 50 cm. The quality control procedures have been drafted by the Waterschapshuis, and executed by the companies NEO and Geodelta. The quality control consists of more than 40 procedures, including the point density check, the filtering of vegetation, the point distribution and the quality of the strip adjustment.

The quality control procedures are partly revised and modified every year. In 2008 the quality measures were derived by comparing corresponding elements in strip overlaps, but expressed for the entire coverage area. Since 2009 the quality measures are estimated and expressed per individual strip overlaps. The quality control procedures are not based on a standard methodology, and in fact there is a lack of reliable and commonly accepted methods for the quality control of laser altimetry data in practice.

## 3. Strip Adjustment Using Planar Features

**Figure 2.**
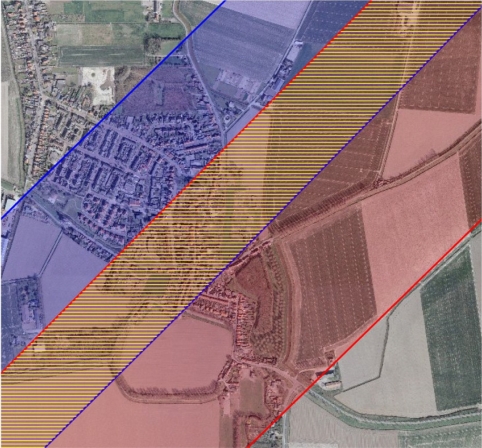
Overlapping strips. The overlap area between the blue strip and the red strip is represented as the yellow hatched area.

The airborne laser scanner data are normally acquired strip-wise, with across-track overlap, as shown in Figure 2. In an ideal dataset that is completely free of systematic errors corresponding planar features in the overlapping area should match perfectly. In practice, this is often not the case, and the distances between the planar features in the two strips can be used to evaluate the systematic and random errors in the data. The following sections describe the procedure for extracting planar features from laser data and the least-squares model for the estimation of the strip adjustment parameters.

### 3.1. Extraction of Planar Features from Aerial Laser Data

Extracting planar features from laser altimetry data is usually done over urban areas where many buildings with planar roofs are available. There are many methods in the literature for detecting buildings and identifying roof planes in aerial laser data, e.g., [27–32]. In this study, a region growing segmentation method is used to extract planar features from gabled roofs or dike slopes. The points that belong to each strip in the overlapping area are interpolated into a raster format with a resolution of 50 centimeters using an Inverse Distance Interpolation algorithm based on the weighted average method [33]. Slope and aspect orientation that best define the terrain in the vicinity of each point are derived. Homogenous regions are created by using a segmentation algorithm performed with the slope and aspect data. The size of the created regions is dependent on a scale parameter, which defines the maximum allowed heterogeneity within the resulting regions. The region-growing segmentation starts with single data points as initial regions, and repeatedly merges them into larger regions as long as a homogeneity criterion holds. The homogeneity criterion is controlled by the scale parameter: larger scale parameters result in larger regions, and smaller parameters in smaller regions [34,35]. To avoid overgrown regions, a suitable scale parameter is found experimentally. The segmentation is performed with different scale parameters on a subset of data, and the segmentation results are visually examined. The best scale parameter is selected as one that provides the largest regions which do not overgrow beyond the boundaries of the roofs or dike slopes.

Planar surfaces that represent gabled roofs and dike slopes are selected from the resulting regions by constraining the regions to a minimum area of 6 m^2^ and a slope between 15 and 70 degrees. The boundaries of the corresponding regions in the two strips are combined and intersected. These intersected regions are buffered inwards with 25 cm (half the raster pixel size) to make sure that the points that are within the intersection area are part of the plane. These points are selected for the estimation of the plane parameters. As the segmentation is performed on gridded data, the selection criteria are not applied on the points directly. Therefore outliers in the selected point cloud might still be present after the segmentation process.

### 3.2. Robust Plane Fitting Using RANSAC

The plane parameters are obtained by applying the Principal Component Analysis (PCA) to the selected points [36]. The principal components are the axes of maximum variation of the data. For a set of points that lie on a plane the last principal component is the axis of minimum variation, which is normal to the plane. The principal components are found by the Singular Value Decomposition (SVD) of the mean-centered points. The eigenvector corresponding to the smallest eigenvalue is the last principal component that is the plane normal. The distance of the plane to the origin of the coordinate system is taken as the median of the dot product of the normal vector and the vector of each individual point.

**Figure 3.**
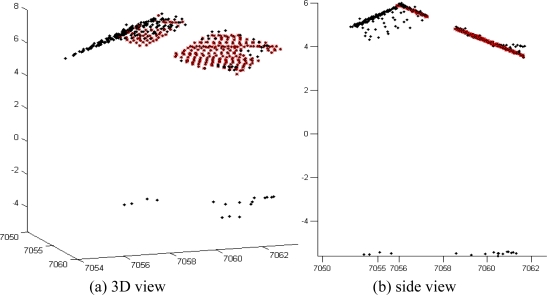
Robust plane fitting to points on a roof segment. Points marked in red are inliers identified by RANSAC.

The segmentation procedure does not necessarily always provide regions of points that are perfectly coplanar. In practice the regions might contain outliers, e.g., points on the walls, trees or the ground surface. To deal with the outlying points a robust plane fitting has to be applied to the points in both strips. Then only those points that are identified by the robust fitting algorithm as inliers are used to obtain the point-to-plane distances. The robust plane fitting is performed using the RANSAC algorithm [37]. Considering that each random sample should contain three points (minimum to define a plane), and assuming that 50% of the points are outliers, 35 random samples are needed to ensure, with a probability of 99%, that at least one sample is outlier-free [38]. The relatively small number of the required random samples indicates that the computational cost of the robust plane fitting is easily affordable. Figure 3 demonstrates the robust plane fitting using RANSAC.

### 3.3. Estimation of Strip Adjustment Parameters

The output of the previous step is a set of planes in one strip and their corresponding points in the other strip. The parameters of a transformation between the two overlapping strips are estimated by minimizing the distances between points and their corresponding planes. Let the transformation of a set of points **P** by a transformation **H** be expressed by matrix multiplication **H P**. The condition that the transformed points lie on a plane **Л** is expressed as [38,39]:

(1) $${Л}^{T}\mathrm{HP}=0$$

where **Л** = (*n_1_*, *n_2_*, *n_3_*, *−d*)^T^ represents a plane with normal **n** = (*n_1_*, *n_2_*, *n_3_*)^T^ and distance *d* to the origin, **P** = (*x*, *y*, *z*, *1*)^T^ denotes the homogenous representation of a point in 3D space. In practice, **H** can be a homogenous similarity transformation:

(2) $$H=\left[\begin{array}{ll} R & t\\ 0_{1\times 3} & 1 \end{array}\right]$$

in which **R** is a 3D rotation matrix, **t** = (*t_x_*, *t_y_*, *t_z_*)^T^ is a translation vector and a scale factor is neglected since laser strips are normally assumed to have identical scales. However, to make the estimation model linear, we ignore the orthogonality of R and assume that **H** is an affine transformation. To estimate the transformation Equation (1) is rewritten as:

(3) $$\left[\begin{array}{llll} n_{1} & n_{2} & n_{3} & -d \end{array}\right]\left[\begin{array}{ll} r_{1}^{T} & t_{x}\\ r_{2}^{T} & t_{y}\\ r_{3}^{T} & t_{z}\\ 0_{1\times 3} & 1 \end{array}\right]\left[\begin{array}{l} p\\ 1 \end{array}\right]=0$$

where **r**_1_^T^, **r**_2_^T^, **r**_3_^T^ are the three rows of **R**, and **p** = (*x*, *y*, *z*)^T^ is the Euclidian notation of a point in 3D space. For one point on one plane Equation (3) reduces to:

(4) $$n_{1}\left(r_{1}^{T}p+t_{x}\right)+n_{2}\left(r_{2}^{T}p+t_{y}\right)+n_{3}\left(r_{3}^{T}p+t_{z}\right)=d$$

Equation (4) is the essential observation equation that basically expresses the condition that the transformed points in one strip rest on their corresponding plane in the other strip. For *m* points corresponding to *k* planes we will have a system of equations as follows:

(5) $$\left[\begin{array}{cccccc} n_{1}^{1}p^{1^{T}} & n_{2}^{1}p^{1^{T}} & n_{3}^{1}p^{1^{T}} & n_{1}^{1} & n_{2}^{1} & n_{3}^{1}\\ \vdots & \vdots & \vdots & \vdots & \vdots & \vdots \\ n_{1}^{k}p^{m^{T}} & n_{2}^{k}p^{m^{T}} & n_{3}^{k}p^{m^{T}} & n_{1}^{k} & n_{2}^{k} & n_{3}^{k} \end{array}\right]\left[\begin{array}{l} r_{1}\\ r_{2}\\ r_{3}\\ t_{x}\\ t_{y}\\ t_{z} \end{array}\right]=\left[\begin{array}{l} d^{1}\\ \vdots \\ d^{k} \end{array}\right]$$

where superscripts denote the point and plane numbers. The system of Equation (5) is of the form AX = L for which the least-squares solution that minimizes the norm of the squared distances between the transformed points and their corresponding planes, *i.e.*, ||AX − L||, is given in Equation (6):

(6) $$X={\left(A^{T}\mathrm{WA}\right)}^{-1}A^{T}\mathrm{WL}$$

where **W** is a weight matrix. In this paper, the same weight is applied to each observable based on the assumption that all observables have the same precision.

A special case of the strip adjustment model as described above occurs when we expect the transformation between the strips to be only a translation vector. In such a case, the estimation model given in Equation (4) reduces to:

(7) $$n_{1}(x+t_{x})+n_{2}(y+t_{y})+n_{3}(z+t_{z})=d$$

which basically expresses that the translated points lie on the plane. The system of equations with *m* points and *k* planes becomes:

(8) $$\left[\begin{array}{ccc} n_{1}^{1} & n_{2}^{1} & n_{3}^{1}\\ \vdots & \vdots & \vdots \\ n_{1}^{k} & n_{2}^{k} & n_{3}^{k} \end{array}\right]\left[\begin{array}{c} t_{x}\\ t_{y}\\ t_{z} \end{array}\right]=\left[\begin{array}{c} d^{1}-n_{1}^{1}x^{1}-n_{2}^{1}y^{1}-n_{3}^{1}z^{1}\\ \vdots \\ d^{k}-n_{1}^{k}x^{m}-n_{2}^{k}y^{m}-n_{3}^{k}z^{m} \end{array}\right]$$

for which a solution that minimizes the distances between the translated points and their corresponding planes can be obtained similar to the general case as given in Equation (6).

It is worth noting that to obtain a solution for the estimation models derived above the design matrices in Equations (5) and (8) have to be of full rank. This requires a minimum of 12 points and 3 non-parallel planes to estimate the affine transformation in Equation (5), and a minimum of 3 points and 3 non-parallel planes to estimate the translation vector in Equation (8). In practice, this is not an issue since usually a large number of point-to-plane distances are available.

The precision of the observables as well as the estimated transformation parameters are derived from the residual vector **v** that actually contains the remaining point-to-plane distances after the adjustment:

(9) v=AX−L

The reference variance obtained from the residual vector **v** is an indication of the precision of the observables, or the random error of individual points:

(10) $$\sigma_{0}^{2}=\frac{v^{T}\;\;W\;\;v}{m-u}$$

where *u* is the number of unknown parameters in the adjustment. The precision of the estimated transformation parameters is then obtained by:

(11) $$\Sigma_{\mathrm{xx}}=\sigma_{0}^{2}{\left(A^{T}\;W\;A\right)}^{-1}$$

## 4. Results of Accuracy Assessment of AHN-2 Data

### 4.1. Description of Dataset

**Figure 4.**
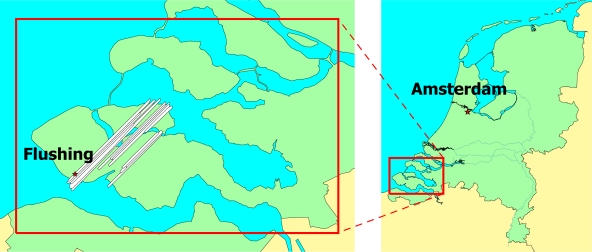
AHN-2 laser altimetry dataset of Zeeland consisting of 15 strips.

The strip adjustment method as described above was employed to assess the accuracy of the pilot AHN-2 dataset acquired by Fugro-Inpark over Zeeland province in 2007. The area consists of a crop land with large farm buildings and several urban areas, of which the city of Flushing is the largest. The data were acquired by the helicopter-mounted FLI-MAP 400 laser scanner from an altitude of 375 meters. The data consists of the forward-, nadir- and backward-looking scan lines. From this dataset, 15 strips were selected, resulting in 13 overlaps, as shown in Figure 4. Table 1 lists the overlaps and their corresponding strips, number of points and width of each overlap. The overlap areas are of different sizes. Within each overlap also the numbers of points belonging to each strip are slightly different, which is due to the difference in the scanning geometry, the topography and the type of objects present in the overlap area.

### 4.2. Evaluation of Segmentation

**Figure 5.**
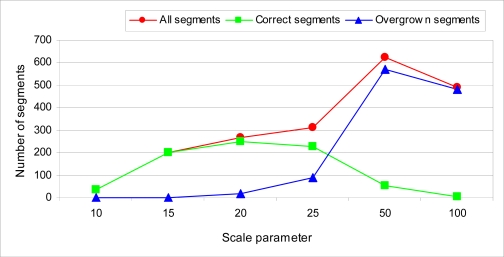
Performance of the segmentation method with different scale parameters.

To evaluate the performance of the segmentation procedure strip overlap o12 was segmented with different scale parameters. The resulting roof regions were examined and compared with building boundaries from an existing large scale base map and with aerial images of the area. The result is shown in Figure 5. Scale parameters 10 and 15 provide the most reliable roof segments. Scale parameter 15 provides more segments that are also larger in size; therefore, it is chosen as the optimal scale parameter.

**Figure 6.**
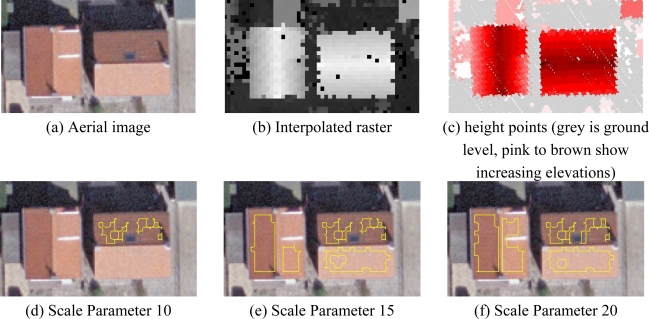
Roof segments created by using different scale parameters in the segmentation procedure.

Figure 6 shows an example of roof segments created by using three different scale parameters. The segments are shown in yellow outlines. The building on the left has a dormer on the right roof side. It can be seen that by using scale parameter 20 one of the created segments is overgrown and contains points on the dormer. In contrast, scale parameter 15 provides homogeneous segments within the roof boundaries. The irregularity in the shape of the segments is due to the intersection of the segments in the two strips and the applied inwards buffering.

### 4.3. Accuracy Assessment Results

The point-to-plane distances were derived for each strip overlap from the segmentation results. For every region in one strip plane parameters were computed using the robust plane fitting method. Then, distances between this plane and the inlying points in the corresponding region in the other strip were obtained and entered as observables in the estimation model. Two transformations were estimated for each pair of overlapping strips: a 3D translation only and a full affine transformation.

**Figure 7.**
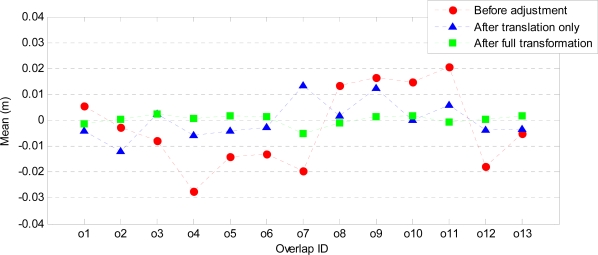
Mean of point-to-plane distances before and after the strip adjustment.

**Figure 8.**
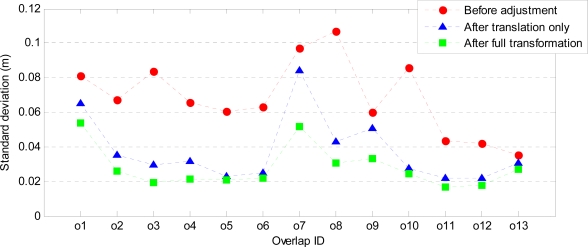
Standard deviation of point-to-plane distances before and after the strip adjustment.

The mean and standard deviation of the point-to-plane distances before and after the adjustment were computed. Assuming that the data are not contaminated by gross errors (outliers), the mean before the adjustment indicates systematic error in the data. After the adjustment, the mean is expected to be very small, and the standard deviation indicates the random error of the observables and the precision of the estimated parameters. Figure 7 shows the mean of the point-to-plane distances before and after the adjustment. Before the adjustment the mean ranges from −3 cm to 2 cm. Adjustment with only a translation reduces the mean in all overlaps, except for overlap o2 (although here the mean is only −1 cm). Adjustment with a full transformation further reduces the mean to values below ±5 mm. Figure 8 shows the standard deviation of the point-to-plane distances before and after the adjustment. Before the adjustment the standard deviations range from 3 cm to 11 cm. These become smaller after the translation (2 cm to 8 cm), and are the smallest after the full transformation where the largest standard deviation is 5 cm.

**Figure 9.**
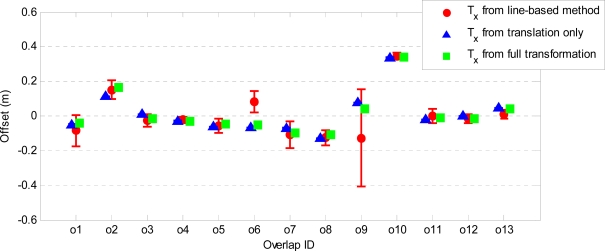
Estimated x-offsets and their associated precision (vertical bars) compared to those estimated from line correspondences.

**Figure 10.**
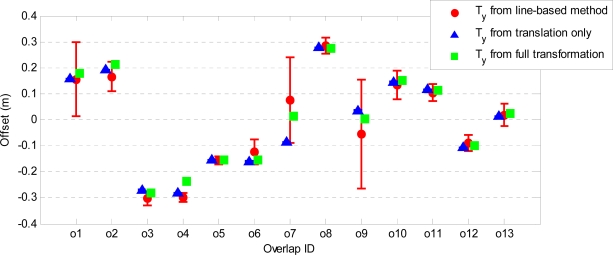
Estimated y-offsets and their associated precision (vertical bars) compared to those estimated from line correspondences.

Figure 9 and Figure 10 show the estimated horizontal offsets respectively in x and y direction together with their precision for all strip overlaps. These values are compared to those obtained by a line-based method [40], which provides only 2D translation parameters. The precision of the estimated parameters is remarkably better in all overlaps when point-to-plane distances are used. Both T_x_ and T_y_ are estimated with a precision better than 1 mm. In most of the overlaps, the estimated horizontal offsets are in agreement with those obtained by the line-based method. The discrepancies are in overlaps o6 (T_x_), o7 (T_y_) and o9 (both T_x_ and T_y_), where differences from 10 cm to 20 cm can be observed. In the case of overlap o9, the precision of both T_x_ and T_y_ estimated from the line correspondences is very low, whereas the offsets estimated from the point-to-plane distances using both adjustment models are more precise and very close in magnitude. In fact, both T_x_ and T_y_ estimated from point-to-plane distances are within the range of uncertainty of the offsets estimated by the line-based method. In overlap o6 also the T_x_ values estimated by the plane-based method using both adjustment models are very close and more precise than that estimated by the line-based method. The disagreement in T_y_ corresponding to overlap o7 may be due to the presence of a small rotation between the strips. Note that the mean and standard deviation of the point-to-plane distances after the adjustment with a translation only and those after the full affine transformation have the largest discrepancy in overlap o7 (see Figure 7 and Figure 8).

**Figure 11.**
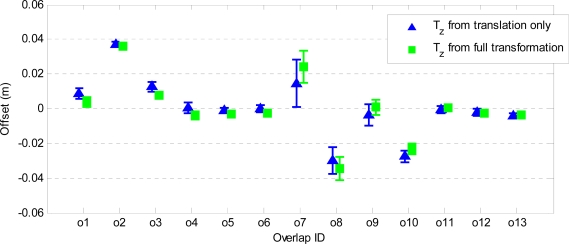
Vertical offsets and their associated precision (vertical bars) estimated from point-to-plane distances.

Figure 11 shows the vertical offsets T_z_ estimated from the point-to-plane distances for all overlaps. Here the bars representing the precision of the offsets are magnified by a factor of 10. It can be seen that the vertical offsets resulting from the two transformation models are very close. A difference of about 1 cm can be observed in overlap o7, which again might imply the presence of a small rotation between the strips. The precision of the estimated vertical offsets is better than 2 mm.

## 5. Conclusions

We have presented a method for the adjustment and accuracy assessment of airborne laser altimetry strips using planar features. The choice of planar features is particularly appropriate for the accuracy assessment of AHN-2 data over the Netherlands, because buildings with gable roof planes are present almost in all parts of the country, and also the high point density of the AHN-2 data is a determining factor in the increased reliability of the extracted planes. The method is based on estimating a transformation between two overlapping strips by minimizing the distances between points in one strip and their corresponding planes in the other. The accuracy assessment of the AHN-2 laser dataset over Zeeland, the Netherlands, was carried out using the proposed plane-based method, and the results were compared with those of a previously-used line-based method. The results show vertical offsets of up to 4 cm between the overlapping strips, and horizontal offsets ranging from 2 cm to 34 cm.

In comparison with the line-based methods, the use of planar features in strip adjustment leads to a more precise estimation of the transformation parameters. The performance of the plane-based methods is, however, dependent on the reliability of the extracted planes. Despite careful selection of the segmentation parameters we found many segments that contained outliers. The robust plane fitting using RANSAC proved very effective in removing these outlying points. In conclusion, for the plane-based strip adjustment method to be used in practice for the quality control of laser altimetry data robust and reliable plane extraction algorithms are of great importance.

## Figures and Tables

**Table 1.** Specifications of the 13 strip overlaps.

**Overlap ID**	**Strip**		**Number of points in the strip overlap**		**Overlap Width (m)**

	**1st**	**2nd**	**1st**	**2nd**	
o1	s3	s4	21,731,922	27,885,585	200
o2	s4	s5	23,114,236	28,984,667	175
o3	s5	s6	28,984,667	51,271,482	240
o4	s7	s8	24,693,624	24,953,061	175
o5	s9	s8	24,534,357	22,834,121	175
o6	s9	s10	35,156,934	22,427,804	240
o7	s10	s11	10,303,345	7,590,510	200
o8	s12	s13	24,771,459	19,671,036	240
o9	s14	s13	3,267,309	4,177,717	525
o10	s15	s13	22,910,115	14,855,334	240
o11	s2	s1	4,618,326	5,251,392	485
o12	s2	s3	4,618,326	6,164,108	485
o13	s1	s3	65,247,718	77,060,363	320
